# Development of Pharmacological Strategies with Therapeutic Potential in Ischemic Stroke

**DOI:** 10.3390/antiox12122102

**Published:** 2023-12-12

**Authors:** Alejandro Escobar-Peso, Emma Martínez-Alonso, Jaime Masjuan, Alberto Alcázar

**Affiliations:** 1 Department of Research, Hospital Universitario Ramón y Cajal, Instituto Ramón y Cajal de Investigación Sanitaria (IRYCIS), 28034 Madrid, Spain; alberto.alcazar@hrc.es; 2 Department of Neurology, Hospital Universitario Ramón y Cajal, Instituto Ramón y Cajal de Investigación Sanitaria (IRYCIS), 28034 Madrid, Spain; 3 Department of Neurology, Facultad de Medicina, Universidad de Alcalá, 28871 Alcalá de Henares, Spain

**Keywords:** antioxidant compounds, ischemic stroke, neuroprotection, oxidative stress

## Abstract

Acute ischemic stroke constitutes a health challenge with great social impact due to its high incidence, with the social dependency that it generates being an important source of inequality. The lack of treatments serving as effective neuroprotective therapies beyond thrombolysis and thrombectomy is presented as a need. With this goal in mind, our research group’s collaborative studies into cerebral ischemia and subsequent reperfusion concluded that there is a need to develop compounds with antioxidant and radical scavenger features. In this review, we summarize the path taken toward the identification of lead compounds as potential candidates for the treatment of acute ischemic stroke. Evaluations of the antioxidant capacity, neuroprotection of primary neuronal cultures and in vivo experimental models of cerebral ischemia, including neurological deficit score assessments, are conducted to characterize the biological efficacy of the various neuroprotective compounds developed. Moreover, the initial results in preclinical development, including dose–response studies, the therapeutic window, the long-term neuroprotective effect and in vivo antioxidant evaluation, are reported. The results prompt these compounds for clinical trials and are encouraging regarding new drug developments aimed at a successful therapy for ischemic stroke.

## 1. Introduction

Ischemic stroke is characterized by an occlusion of a brain blood vessel, which impairs cerebral blood flow levels. The consequent decrease in oxygen and nutrients and the dysfunction of toxic metabolite elimination trigger a set of metabolic responses within the brain cells, known as ischemic damage, that endangers the cerebral tissue and may eventually lead to infarction. The latest Global Health Estimates report by the World Health Organization in 2019 cited stroke as the second leading cause of death worldwide, accounting for approximately 11% of the total (more than 6 million people), and the third leading cause of disability [1]. Ischemic stroke represents approximately 80% of all stroke cases; therefore, new therapies aimed at treating this disease are urgently required.

Currently, clinical therapeutic approaches used for ischemic stroke are aimed at the recanalization of the occluded vessel and the reperfusion of the compromised cerebral tissue. These approaches include pharmacological dissolution (thrombolysis) and mechanical removal (thrombectomy) of the thrombus [2,3]. Reperfusion is an essential contributor to stroke-related patient outcomes, as it allows for the recovery of normal cerebral blood flow levels and avoids the progression of the infarction core. However, it is also considered a double-edged sword because re-oxygenation during reperfusion exacerbates ischemic damage due to the induction of oxidative stress [4], thus contributing to additional neuronal death and constituting ischemia–reperfusion damage [5].

As a consequence of occlusion and nutrient deprivation during the acute ischemic phase, compromised tissue undergoes functional and structural alterations, thus making it susceptible to radical species attacks and further damaging cell structures, including membranes, proteins or nucleic acids [5]. In addition to this set of processes triggered during vessel occlusion, known as ischemic cascade, the recovery of normal blood flow levels—i.e., reperfusion—in the compromised tissue exacerbates some of the damaging components of the cellular response to the ischemic insult, which further compromises the fate of brain cells [5]. Oxidative stress after the ischemia–reperfusion damage is driven by an overproduction of radical species that overwhelms the endogenous antioxidant properties of the brain cells. Edaravone (Radicut^®^), authorized in Japan in 2001 [6,7] and also used in China [8,9] and India [10], and *N*-butylphtalide, obtained from celery and approved in China in 2002 [11], are the only examples of ischemia–reperfusion damage-directed therapies, but they have not yet been granted approval by regulatory agencies, such as the FDA or EMA, for the treatment of ischemic stroke.

This lack of adequate treatment has led to extensive research in recent decades. From ionic homeostasis regulation [12,13] to inflammation modulation [14,15] or oxidative stress attenuation [16], among others, different strategies have arisen with the aim of protecting brain cells after the ischemic insult. Frequently, these new candidate therapies have been developed as complementary to the recanalization approaches mentioned previously, as the recovery of normal blood flow levels, induced either clinically or spontaneously, is a required feature before any other therapeutic approach can be used.

**Scheme 1:**
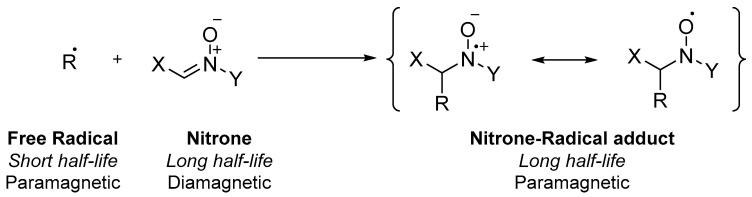
Nitrones, as radical spin traps, originally developed for electronic paramagnetic resonance (EPR) studies. The labeling of “short” and “long” half-lives in the scheme refers to their ability to be detected via EPR studies.

In this context, the development of new and powerful antioxidant drugs remains of crucial interest. Antioxidant effects can be exerted by compounds that (i) scavenge free radicals, avoiding radical chain reactions against relevant cell structures, (ii) activate endogenous antioxidant systems, (iii) replenish depleted intracellular antioxidant pools, or (iv) avoid the malfunctioning of cell systems that are producers of radical stress. Chemical compounds with a *N*-oxide of the imine group (nitrone) were first developed as radical traps that allowed for the detection of unstable and short-lived free radicals in electronic paramagnetic resonance (EPR) techniques via the formation of corresponding reaction products with longer half-lives, thus making them more easily detected (Scheme 1). Since then, and with the increasing knowledge of the pathophysiology of oxidative stress, their potential as candidates for the treatment of ischemic stroke and related diseases has been rapidly prompted [17,18].

Years of developing nitrone-derived compounds have led to NXY-059 (Cerovive^®^, OKN-007), a phenyl *N*-*tert*-butyl nitrone (PBN) derivative, which constitutes the most advanced nitrone in clinical development to date. NXY-059 reached phase III clinical trials, but its development was terminated after no significant improvement was concluded [19]. The main causes of this failure were identified as the low permeability of the compound and biases in the in vivo experimental assays in their preclinical development, along with the questionable design of the clinical trials [20,21].

Despite this, the features of the nitrone group, including its great versatility, antioxidant effects and pharmacological safety, have prompted the development of a plethora of nitrone-derived structures for the treatment of ischemic stroke and other diseases characterized by cell damage through radical formation.

It is widely known that the radical trapping activity is granted via the nitrone moiety itself, in contrast to other natural antioxidants [22,23]. The capacity to identify a precise structural motif as the cause of its radical scavenging behavior makes it possible to attach it to any other main scaffold, easily conferring spin trapping activity. In this regard, the conception of nitrone-derived compounds, combining a nitrone and a scaffold serving as a core, allows the implementation of new functionalities provided by the main structure. Additionally, it allows the modulation of the activity and the pharmacological properties via changes to the main scaffold, its connectivity with the nitrone group and the type and position of its substituents. This provides new possibilities for the design of drug candidates that might retain a function based on the main scaffold, along with radical trapping properties due to their nitrone moiety, such as new pleiotropic drugs. These include complex biomolecules such as lipids, proteins or carbohydrates, giving rise to an interesting field of research in drug compartmentalization [24,25,26,27] or special biological target modulation [28,29].

Apart from this, nitrones may have other applications. Their radical trapping activity, most recently considered a therapeutic feature but originally developed as an analytical attribute, can convert nitrones into potent theragnostic tools that can not only treat diseases but can also increase the knowledge about the pathophysiology of different oxidative-stress-driven diseases. In line with this biological chemistry consideration, the nitrone group also constitutes an example of a biorthogonal moiety. Being easily attachable to almost any molecule, the nitrone group can quickly react in biological settings through cycloaddition reactions [28,29,30], allowing its use across a multitude of applications.

**Figure 1:**
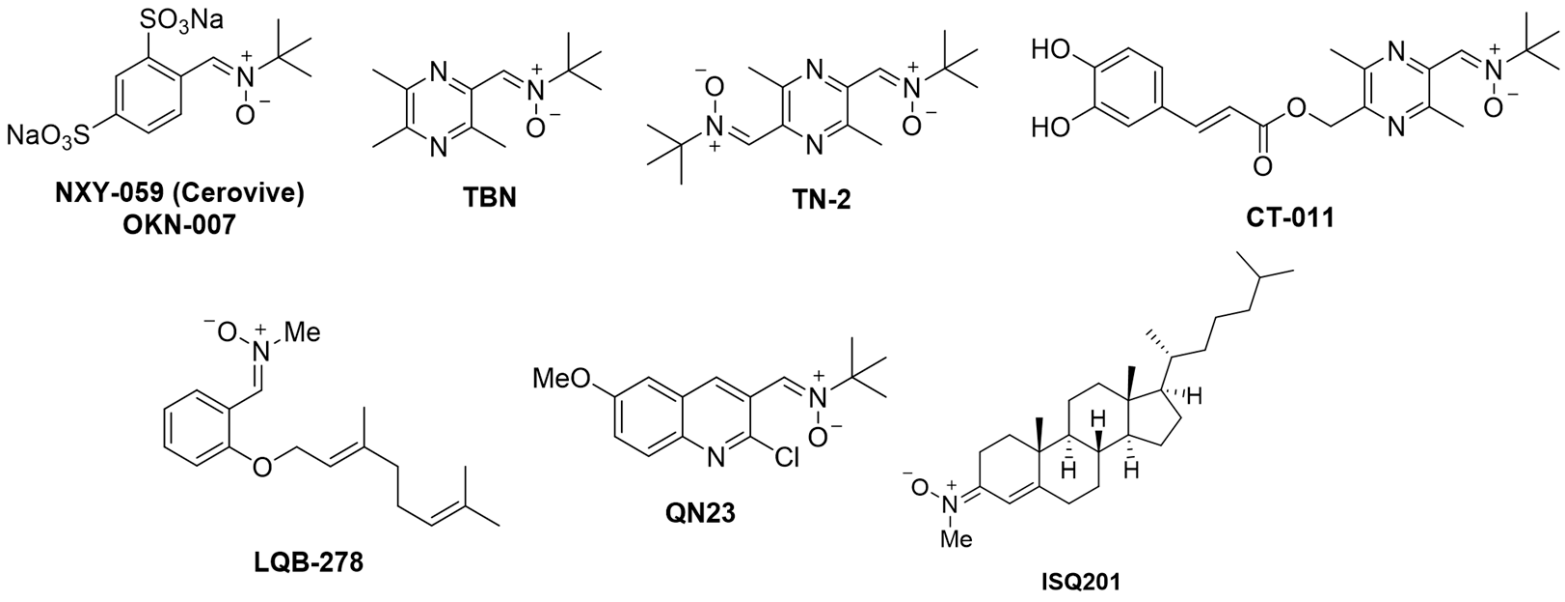
Examples of nitrones currently in development as therapeutic agents.

In Figure 1, we present some examples of nitrones currently being developed as drug candidates for ischemic stroke or other diseases. NXY-059 has been reported to work as a chemotherapeutic agent against glioblastoma [31,32], with several clinical trials being conducted. TBN (tetramethylpyrazine nitrone), derived from 2,3,5,6-tetramethylpyrazine, an active component of the traditional Chinese herb *Ligusticum wallichii*, combines a thrombolytic effect with potent radical scavenger properties [33] and a good permeability profile, proving to be neuroprotective in ischemic stroke [34], Alzheimer’s disease [35] and amyotrophic lateral sclerosis [36] experimental models. Its development has led to some derivatives, such as TN-2 [37] and CT-011 [38], which are also effective in different ischemic stroke experimental models. LQB-278 is a terpenoid–nitrone hybrid currently being studied as an antiproliferative agent in leukemia [39]. Lastly, we included the most advanced nitrones developed by our group to date, the ischemic stroke drug candidates quinolylnitrone QN23 and cholesterol–nitrone ISQ201, which we will thoroughly describe in this review (Figure 1).

## 2. Development of New Pharmacological Neuroprotective Strategies for the Treatment of Ischemic Stroke

In previous years, we have worked on a drug development project aimed at the treatment of ischemic stroke via nitrone-derived structures. In collaboration with the Laboratory of Medicinal Chemistry at IQOG (CSIC, Madrid, Spain), the Neurovascular Research Group (IBIS, Seville, Spain) and the Mixed Unit of Cerebrovascular Research (IIS La Fe, Valencia, Spain), we have evaluated different sets of new nitrone-derived compounds. This review is the summarized development of two drug candidates achieved: quinolylnitrone QN23 and the cholesterol–nitrone hybrid (cholesteronitrone) ISQ201.

**Figure 2:**
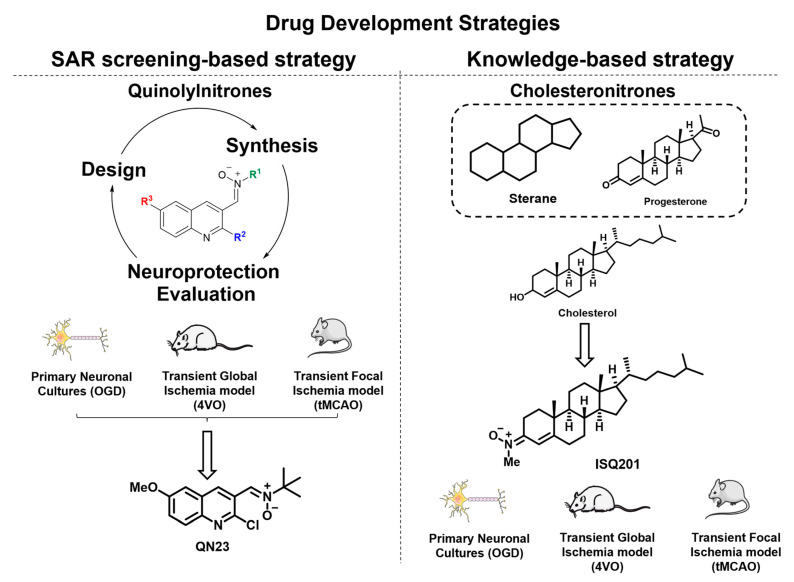
Summarized drug development strategies of QN23 and ISQ201: structure–activity relationship (SAR)-screening-based and knowledge-based strategies, respectively.

These two compounds have been developed in parallel but with different approaches to the drug development process. The study of the quinoline core that led to our lead compound, QN23, followed a “classical” medicinal chemistry approach, based on a reiterative process including the design, synthesis and biological evaluation of a derived set of compounds. It has been named a “hit to lead” process; that is, a structure–activity relationship (SAR)-screening-based strategy (Figure 2). In contrast, the development of cholesterol-derived nitrone ISQ201 arose from the consideration of previous knowledge about neuroprotective molecules, such as progesterone and other steroid derivatives, and their interesting biological activity in the context of ischemic stroke protection (Figure 2).

Despite being the driving force of our initial design, radical trapping activity may not be the only cause of the biological activity found for nitrones. Numerous studies have produced controversy over the feasibility of a radical trap as a therapeutic candidate, at least with no other contributions, in terms of the high concentration required for spin trapping experiments in chemical media, which is much higher than the concentration normally used in biological settings [22]. These findings necessitate the consideration of alternative or complementary modes of action in the biological activity of nitrones that may account for the observed neuroprotective effect.

In the two drug development programs presented herein, special attention is given to the evaluation process. It has mainly been based on phenotypical assays in complex biological systems, such as primary neuronal cultures subjected to oxygen and glucose deprivation (OGD), or animal experimental models, aiming to obtain a comprehensive understanding in the precise assessment of the biological effect of these compounds. In this review, we focus on the biological and pharmacological evaluations of these compounds that have led to our most advanced candidates to date, QN23 and ISQ201.

### 2.1. Quinolylnitrones as a Result of a Screening Program

**Figure 3:**
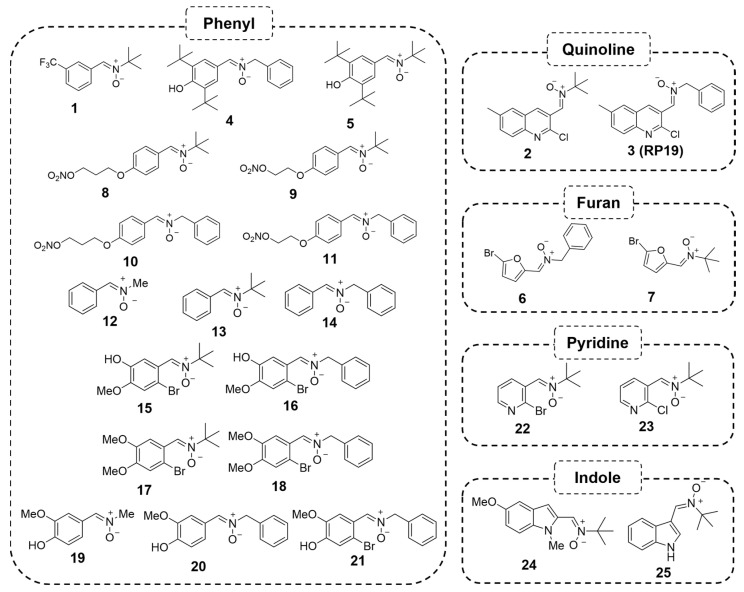
Nitrone-derived compounds synthesized for preliminary scaffold screening in the development of quinolylnitrone drug candidates. Structures of nitrones **1**–**25**, named according to [40].

Our first approach to the development of new nitrone-derived drug candidates consisted of a preliminary screening of different scaffolds serving as cores for the nitrone moiety attachment. All of them were based on simple structures available or designed via Dr. Marco-Contelles’ Laboratory of Medicinal Chemistry at IQOG (CSIC, Madrid, Spain), thus leading to an extended number of products to be screened. The first approach included 25 nitrone compounds derived from phenyl, pyridine, furan, indole and quinoline scaffolds (Figure 3) [40].

The particular choice of the mentioned scaffolds was based on the following: (i) the structural basis of the long-documented nitrone PBN and its derivative NXY-059, which are phenyl-derived, and (ii) the exploration of different heteroaryl-derived scaffolds, which are known to increase the spin trapping activity of the corresponding nitrone and the stability of the spin adduct, as well as to modulate lipophilicity and solubility [41]. The studied heteroaryl scaffolds included six-membered heteroaryl nitrogen structures, such as pyridine, and related bicyclic structures, such as quinolines and indoles, as well as five-membered rings, such as furans.

On the other hand, the choice of the nitrone moiety was based on the structure of PBN (bearing a *N*-*tert*-butyl group), the consideration of low steric-hindering alkyl groups, such as methyl, and the commonly used benzyl. In either case, these substituents were introduced through the corresponding commercially available and easily manageable *N*-substituted hydroxylamine or nitro compound, which are starting materials used in the nitrone formation reaction.

In order to elucidate which structural family possessed the best characteristics for further development as potential candidates for treating ischemic stroke, theoretical calculations, antioxidant assessments and neuroprotection evaluations were carried out. As the most remarkable results, the in vitro assessment of the antioxidant profile revealed a higher tendency to scavenge •OH radicals, as well as an adequate estimated partition coefficient (cLogP) for almost every compound in the set, suggesting a good permeability profile [40]. Regarding the biological characterization, these nitrones were tested in an experimental model of ischemia in primary neuronal cultures subjected to oxygen and glucose deprivation (OGD) in order to assess their neuroprotection in a biological system. The nitrone effect on cell viability was determined via an MTT assay and expressed as neuroprotection when the cell viability observed was over the value obtained for the vehicle-treated cells (R24h value, 0% neuroprotection) with respect to the control cells (100% value).

Among the nitrones tested, a quinolylnitrone (candidate 3, renamed as RP19) exerted the highest neuroprotective effect in primary neuronal cultures subjected to OGD, measured via cell viability [40]. This compound induced a significant neuroprotective value of 105% at 100 µM, possessed a relatively good in vitro antioxidant profile and exhibited a calculated cLogP (4.27) and permeability that would allow this molecule to cross the blood–brain barrier [40]. Therefore, we decided to further characterize the pharmacological and biological effects of RP19 as a potential candidate for ischemic stroke treatment.

#### 2.1.1. Biological Characterization of RP19: A First-Hit Quinolylnitrone

The following biological characterization of RP19 included the assessment of the neuroprotective effect after delayed administration and the determination of its antioxidant capacity via the quantification of its effect on reactive oxygen species (ROS) levels and lipid peroxidation in primary neuronal cultures subjected to OGD [42].

**Figure 4:**
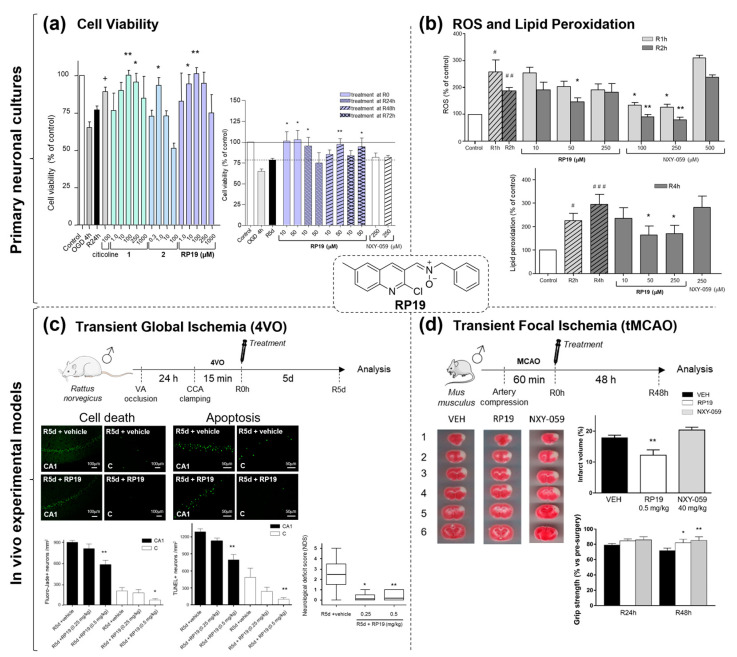
Biological characterization of the compound RP19 as a candidate for the treatment of ischemic stroke. (**a**) Left bar graph: RP19 preserved cell viability in primary neuronal cultures subjected to oxygen and glucose deprivation (OGD) when administered at the onset of the recovery period (R0h). Cell viability of cells treated with RP19 at 10 µM or 100 µM was significantly increased when compared to the cell viability of the vehicle-treated group. * *p* < 0.05 and ** *p* < 0.01 compared with the R24h value by Dunnett’s post-test after ANOVA. Right bar graph: RP19 at 10 or 50 µM preserved cell viability when administered at delayed times at R24h, R48h and R72h after post-OGD recovery onset, as compared to the cell viability of the untreated group at 5 days post-OGD recovery. * *p* < 0.05 and ** *p* < 0.01 compared with the R5d value (dotted line) by Dunnett’s post-test after ANOVA. (**b**) Effect of RP19 on ROS and lipid peroxidation levels in primary neuronal cultures exposed to OGD. Upper bar graph: RP19 at 50 µM decreased ROS levels in primary neuronal cultures subjected to OGD when administered at the onset of the recovery period. ROS levels were evaluated at R2h post-OGD recovery. Lower bar graph: RP19 at 50 µM or 250 µM decreased lipid peroxidation levels in primary neuronal cultures subjected to OGD when administered at the onset of the recovery period. Lipid peroxidation levels were evaluated at R4h post-OGD. # *p* < 0.05, ## *p* < 0.01 and ### *p* < 0.001 for R1h, R2h or R4h compared with their control by the one-sample t test. * *p* < 0.05 and ** *p* < 0.01 compared with R1h, R2h or R4h values by the *t* test. (**c**) Effect of RP19 on neuronal death and apoptosis and the neurological deficit score (NDS) in ischemic rats treated with RP19 at the onset of reperfusion. RP19 at 0.5 mg/kg significantly decreased cell death (35% and 70% for the hippocampal CA1 and cortical regions, respectively), evaluated using Fluoro-Jade B (left image set and bar graph), and apoptosis-positive cells (40% and 80% for CA1 and C, respectively) evaluated using the TUNEL assay (right image set and bar graph) at 5 days of recovery (R5d) after transient global cerebral ischemia via four-vessel occlusion (4VO, vertebral arteries (VA) and common carotid arteries (CCA)). * *p* < 0.05 and ** *p* < 0.01 compared with their respective R5d + vehicle by Dunnett’s post-test after ANOVA. RP19 also decreased NDS at R5d when administered at 0.25 or 0.5 mg/kg. * *p* < 0.05 and ** *p* < 0.01 compared with R5d + vehicle by Dunn’s post-test after Kruskal−Wallis’ ANOVA test. (**d**) The effect of RP19 on infarct size and the grip strength test after transient middle cerebral artery occlusion (tMCAO) in a mouse model. Image set and upper bar graph: RP19 decreased 30% of the infarct size in a tMCAO model when administered at 0.5 mg/kg at the onset of reperfusion, evaluated at 48 h after ischemia. * *p* < 0.01 compared with vehicle by Dunnett’s post-test after ANOVA. Right bar graph: RP19 at 0.5 mg/kg preserved the grip strength in animals subjected to tMCAO at 48 h after ischemia. * *p* < 0.05 and ** *p* < 0.01 compared with vehicle (VEH) by Dunnett’s post-test after two-way ANOVA. Figures were reprinted (adapted) with permission from *J. Med. Chem.* and *ACS Chem. Neurosci.* Copyright 2012 and 2017, American Chemical Society [40,42].

Usually, drugs aimed at the treatment of ischemic stroke are tested as preconditioning or at the onset of the post-ischemic recovery period, which, despite providing useful information and easier work methodologies, are difficult or even impossible to translate into clinical practice. The early estimation of the neuroprotective effect of candidates at delayed administration times after the ischemic insult is required in order to fully assess their neuroprotective effect and activity profile in relevant terms for clinical use. Thus, as a preliminary approach to this issue, we decided to explore the effect of a delayed administration of RP19 in primary neuronal cultures at 24, 48 and 72 h after OGD (Figure 4a). Remarkably, even though the highest neuroprotective effect was observed when RP19 was administered at the onset of the recovery period (R0h), the administration of either 10 or 50 µM of RP19 achieved statistically significant higher cell viability at the studied time points than the one observed after 5 days of recovery for the untreated group (Figure 4a) [42].

As was mentioned in the Introduction Section, oxidative stress damage is driven by the overwhelming production of ROS that may damage lipid structures, which, in the brain, are particularly vulnerable to oxidative stress due to their polyunsaturated composition, producing lipid peroxidation. Assessing the effect of RP19 on the levels of ROS and lipid peroxidation was determined as shown in Figure 4b. RP19 significantly decreased ROS levels after 2 h-long treatment at 50 µM, compared to the untreated group. Remarkably, RP19 at 50 or 250 µM significantly decreased the levels of lipid peroxidation, as determined via MDA levels, the maximum of which was observed after 4 h of post-ischemia–reperfusion (Figure 4b) [42].

In an experimental model of transient global cerebral ischemia, the intraperitoneal administration of RP19 at the onset of the reperfusion period was followed by a study of the cell degeneration, apoptosis and neurodeficit score at 5 days of recovery after ischemia (Figure 4c) [42]. The sample size was determined using power analysis (http://www.biomath.info/power/ttest.htm, accessed on 12 March 2020), with the significance level set at 0.05 and statistical power set at 0.8 (80%), rendering a sample size of six animals per group. In this study, 6–8 animals were used for the RP19-treated groups, and 14 animals were used for the vehicle group. This in vivo model was performed with 15 min of ischemia followed by reperfusion, inducing delayed neuronal death in hippocampal *cornu ammonis* 1 (CA1) and various layers of the cortical region. Treatment with RP19 significantly reversed damage produced after the ischemic insult, as indicated in the neurodeficit score (for 0.25 and 0.5 mg/kg doses), cell degeneration via Fluoro-Jade B stain (significant reduction observed at 0.5 mg/kg, in both the CA1 and cortical regions) and cell apoptosis via the TUNEL assay (at 0.5 mg/kg, for both cerebral regions studied) (Figure 4c) [42].

Finally, the effect of RP19 was also determined in a transient focal ischemic stroke model in mice. The same sample size calculation (see above) resulted in 6 animals per group, with 6 and 10 animals treated with RP19 and NXY059, respectively, and 16 animals included in the vehicle-treated group. This experimental model consisted of a 60-min-long occlusion of the middle cerebral artery (tMCAO model), after which animals were intraperitoneally treated and allowed to recover, and the functional deficit and infarct size were analyzed. An analysis of the functional deficit via grip strength assessment carried out 24 and 48 h after the ischemia revealed a significant improvement in RP19-treated animals when compared with untreated animals after 48 h of recovery. The infarct size, measured after 48 h of post-ischemic recovery via TTC stain, indicated a significant reduction in infarct volume in animals treated with RP19 when compared to vehicle-treated animals (Figure 4d) [42].

Although RP19 showed a weak antioxidant effect on ROS levels, this compound showed an effective reduction in lipid peroxidation (Figure 4b), which is an effect of great importance in cerebral ischemia considering the high concentration of polyunsaturated fatty acids in the brain compared to other organs. In addition, it could not be ruled out that unknown targets of RP19 could also participate in its neuroprotective effect, such as metabotropic glutamate receptor 1 and vascular endothelial growth factor (VEGF), identified as RP19 targets in an in silico target-identification study [5]. These proposed targets have obvious roles in excitotoxicity and angiogenesis and are both relevant mechanisms in protection against cerebral ischemia.

These results confirmed the good neuroprotective potential of the RP19 candidate as our first hit, prompting us to further explore the quinoline core in order to find other potential quinolyl-derived drug candidates with adequate biological and pharmacological features for the treatment of ischemic stroke.

#### 2.1.2. Structure–Activity Relationship Study: The Identification of QN23

**Figure 5:**
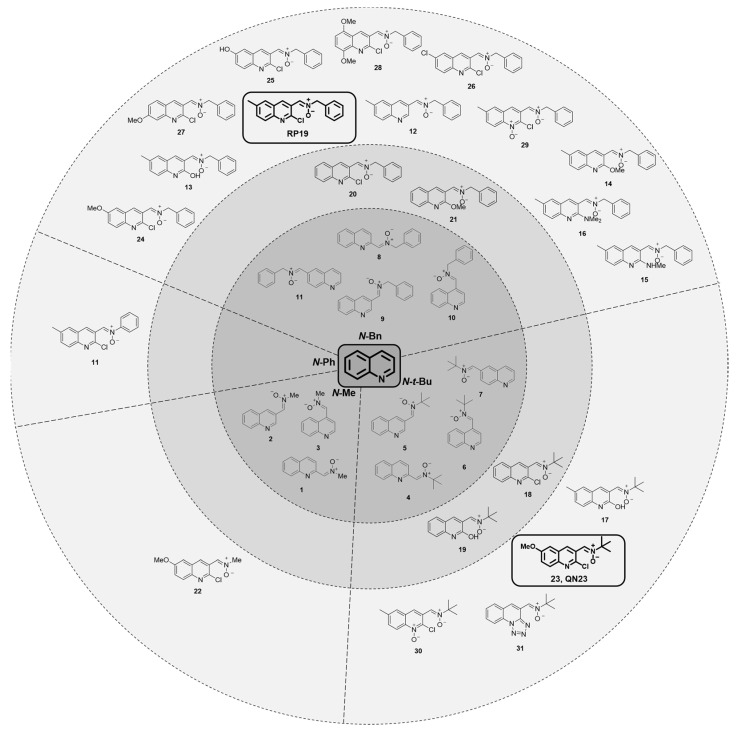
Structure–activity relationship study of quinolylnitrone derivatives. More than 40 compounds were assayed as potential neuroprotective agents for the structure–activity relationship study of quinolylnitrones. Compounds are located in sections depending on the substituent of the nitrone group: benzyl (*N*-Bn), *tert*-butyl (*N*-*t*-Bu), methyl (*N*-Me) or phenyl (*N*-Ph). From the core of “nude” quinoline, compounds are situated in layers depending on the modification of the quinoline core. Compound names for structures in the innermost circle are taken from the original reference [43]. Compound names from the outer circles are taken from the original reference [44].

The in-depth study of the quinoline core as the main scaffold for new nitrone-derived compounds included more than 40 new structures derived from RP19 [43,44]. The nitrone compounds were synthesized and designed by Dr. Marco-Contelles’ Laboratory of Medicinal Chemistry at IQOG (CSIC, Madrid, Spain), as shown in Figure 5. In Figure 5, we have classified these compounds according to their *N*-substituent, in order to clarify the similarity among the structures. In order to first identify the neuroprotective activity of these compounds, considering the coherent results obtained between models for our previous RP19 development, we again used the cell viability determination evaluation in primary neuronal cultures after OGD for screening.

Our first compound set for exploring quinoline derivatives included the examination of the nitrone moiety over the nude quinoline core (innermost circle, Figure 5). This study included the combination of three different *N*-substituents (methyl, *tert*-butyl and benzyl) in three to four different positions over the quinoline core (compounds QN1–QN11) [43]. Cell viability protection by these compounds after OGD revealed QN6, a *N*-*tert*-butyl quinolylnitrone at position C4, as the most interesting one of the set, with the highest cell viability found [43]. Nevertheless, its effect was not superior to our hit compound RP19, which is why we decided to focus on this structure—substituted at C2 and with the nitrone group over the C3 position—in our next structure–activity relationship studies.

In the ensuing studies, we explored different combinations of the following structural features: (i) the substitution of the nitrone group using other azo-compounds (not shown in Figure 5), (ii) the *N*-substituent of the nitrone moiety, (iii) substituents at the C2, C5, C6, C7 and C8 positions of the quinoline core and (iv) miscellaneous compounds [44]. These structures can be seen in the medium and outermost circles of Figure 5.

An evaluation for a comprehensive understanding of cell viability preservation after treatment with these compounds in an OGD experimental model revealed several crucial aspects. First, no azo-compounds tested (oxime, hydrazone and imine), or the parental carbaldehyde, showed a higher neuroprotective effect than the corresponding nitrones. Second, an analysis of the *N*-substituent in the nitrone group revealed that either *N*-*tert*-butyl or *N*-benzyl generally exerted better cell viability preservation than the corresponding *N*-methyl or *N*-phenyl counterparts. Finally, no clear conclusion was inferred regarding the influence of the substituent on the quinoline core, nor regarding their electronic characteristics or their position [44]. Remarkably, a (C3)-*N*-*tert*-butyl nitrone substituted with a methoxy group at C6 and a chlorine atom at C2 (compound 23 or QN23) afforded the highest neuroprotective activity of the whole set, reaching higher levels than our previous hit compound RP19 [39].

**Figure 6:**
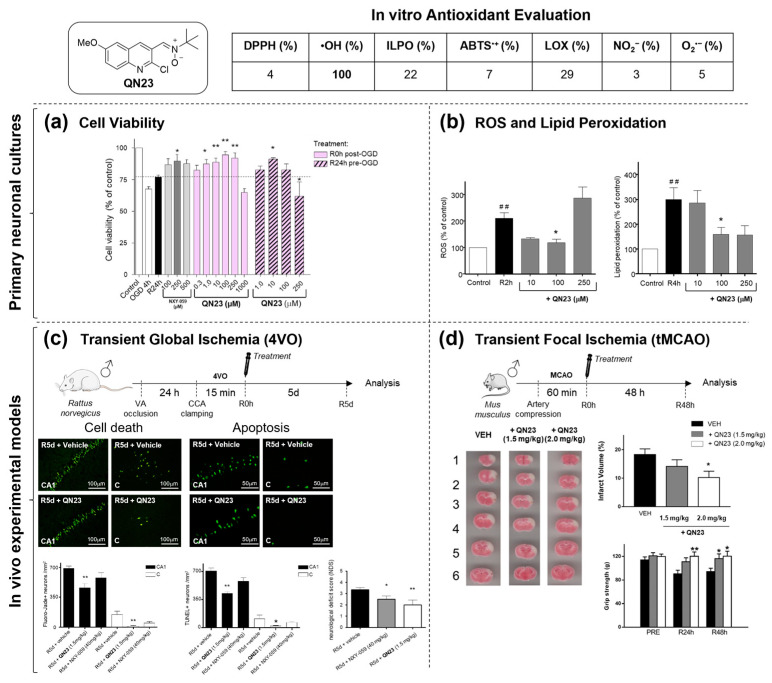
Biological characterization of the QN23 compound as a candidate for the treatment of ischemic stroke. Table: in vitro antioxidant profile of QN23. (**a**) QN23 preserved cell viability in primary neuronal cultures exposed to oxygen and glucose deprivation (OGD) when administered in the range of 1.0–250 µM at the onset of the recovery period, or at 10 µM 24 h before the OGD insult, when compared to the untreated cells. * *p* < 0.05 and ** *p* < 0.01, compared to R24h (dotted line) by Dunnett’s post-test after ANOVA. (**b**) Effect of QN23 on ROS and lipid peroxidation levels in primary neuronal cultures exposed to OGD when administered at the onset of the recovery period. Left bar graph: QN23 at 100 µM decreased ROS levels evaluated at R2h post-OGD recovery. Right bar graph: QN23 at 100 µM decreased lipid peroxidation levels evaluated at R4h post-OGD. ## *p* < 0.01, R2h or R4h compared to their control by the one-sample *t* test; * *p* < 0.05, compared with R2h or R4h by Dunnett’s post-test after ANOVA. (**c**) The effect of QN23 on neuronal death and apoptosis and the neurological deficit score (NDS) in animals treated intraperitoneally with QN23 at 1.5 mg/kg at the onset of reperfusion in an experimental model of transient global cerebral ischemia in rats. QN23 at 1.5 mg/kg significantly decreased cell death (33% and 90% for the hippocampal CA1 and cortical regions, respectively), evaluated using Fluoro-Jade B (left image set and bar graph), and apoptosis-positive cells (40% and 80% for CA1 and C, respectively), evaluated using the TUNEL assay (right image set and bar graph) at 5 days of recovery (R5d) after transient global ischemia via four-vessel occlusion (4VO, vertebral arteries (VA) and common carotid arteries (CCA)). * *p* < 0.05 and ** *p* < 0.01, compared to their respective R5d + vehicle by Dunnett’s post-test after ANOVA. QN23 also decreased NDS at R5d when administered at 1.5 mg/kg. * *p* < 0.05 and ** *p* < 0.01, compared to vehicle by Dunn’s post-test after Kruskal−Wallis’ test. (**d**) The effect of QN23 on the infarct size and grip strength test after a model of transient middle cerebral artery occlusion (tMCAO) in mice. Image set and upper right bar graph: QN23 decreased the infarct size in a tMCAO model (22% for QN23 1.5 mg/kg and 44% for QN23 2.0 mg/kg) when administered at the onset of reperfusion, evaluated at 48 h after the ischemic episode and compared to the untreated animals. * *p ≤* 0.05, compared to vehicle by the Bonferroni post-test after ANOVA. Right bar graph: QN23 preserved grip strength in animals subjected to tMCAO when administered at 1.5 mg/kg or 2.0 mg/kg at the onset of the recovery period and evaluated 24 h or 48 h after the ischemic episode. * *p* < 0.05 and ** *p* < 0.01, compared to vehicle treatment (VEH) by the Bonferroni post-test after two-way ANOVA. Figures were reprinted (adapted) with permission from *J. Med. Chem.* Copyright 2019, American Chemical Society [44].

Antioxidant analysis of a selected subset of nitrones among the more than 40 compounds analyzed, comprising examples with high (QN23), intermediate (QN19 and QN24) or low (QN18 and QN22) neuroprotective effects on OGD, included the assessment of their in vitro radical trapping activities against several radical species. As the most remarkable result, QN23 exerted the highest effect on hydroxyl radical scavenging among the subset tested (Figure 6), with the highest calculated lipophilicity (as stated via the cLogP value of 3.49) [44]. Antioxidant determination in primary neuronal cultures was also carried out by determining the ROS and lipid peroxidation levels after the OGD insult with QN23 treatment. As shown in Figure 6b, the obtained results reveal that QN23 significantly reduced ROS and lipid peroxidation levels after OGD insult when treated with 100 µM [44].

In addition, QN23 treatment prior to the OGD worked for the preservation of cell viability, which was severely compromised after the OGD insult (Figure 6a), even though statistically significant differences were only found for 10 µM of QN23 as a pretreatment and with a lower absolute value than the previously mentioned cell viability preservation values with QN23 after OGD (Figure 6a) [44]. All of these results prompted us to determine the in vivo effect of QN23 on inflammation and neuroprotection. For the former, an assessment of QN23’s power in a carrageenan-induced edema in rat paw revealed similar anti-inflammatory activity to the reference compound indomethacin [44].

Neuroprotective evaluation was determined in a transient global cerebral ischemia model, briefly described earlier in this review (Figure 4, above). In this model, QN23 significantly decreased apoptosis-positive cells in the CA1 and cortical regions, with a dose of 1.5 mg/kg after intraperitoneal administration at the onset of post-ischemia–reperfusion (Figure 6c) [44]. The same dose was also effective for a decrease in cell degeneration, as determined using Fluoro-Jade B staining, in both the hippocampal CA1 and cortical regions (Figure 6c). These results seem to properly correlate with the neurodeficit assessment determined after five days of recovery, which was also ameliorated with QN23 1.5 mg/kg. A power analysis with the significance level set at 0.05 and statistical power set at 0.8 (80%) determined a sample size of 6 and 10 subjects for the treated and control groups, respectively, whereas the QN23-treated experimental groups included 6 animals per group and the vehicle control group included 12 animals.

Regarding the transient focal ischemia model, QN23’s effect on the infarct size and motor deficit 24 and 48 h after the ischemic insult was determined. The infarct size measurement revealed that a QN23 intraperitoneal injection at a dose of 2.0 mg/kg significantly decreased the infarct size by 44% (Figure 6d). A significant improvement in the motor-deficit preservation was also observed in animals treated with QN23 both at 1.5 (48 h) and 2.0 mg/kg (24 and 48 h, as shown in Figure 6d) [44]. In this study, the power analysis (see above) determined eight animals per group, all of which were included in each experimental group.

**Figure 7:**
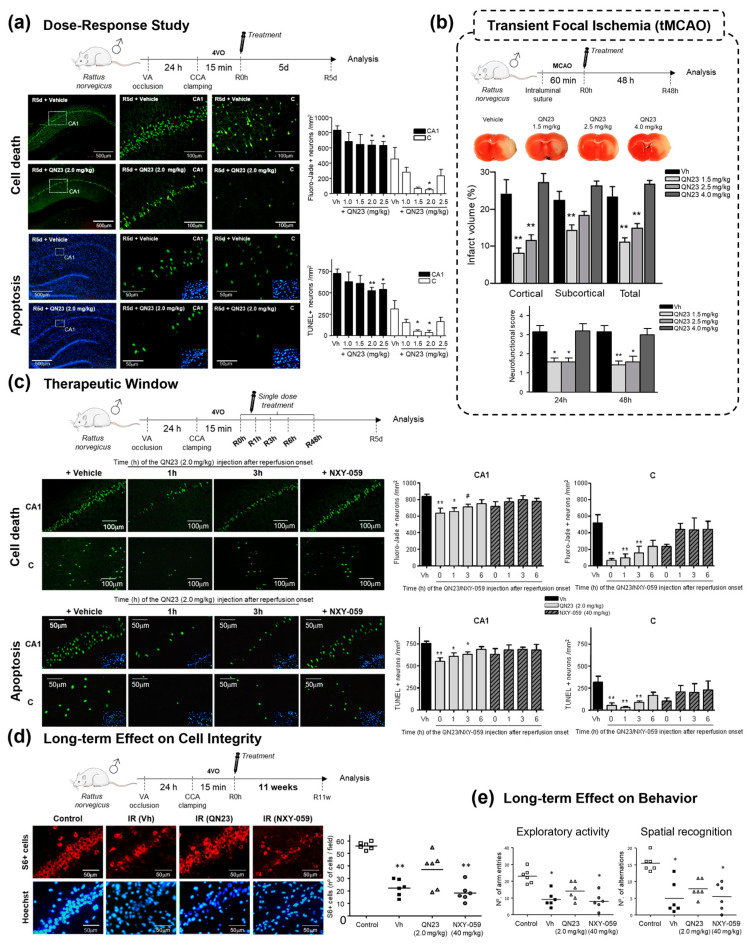
Preclinical characterization of QN23. (**a**) Dose–response curve of QN23 administered intravenously. QN23 significantly decreased cell death (25% and 90% for the hippocampal CA1 and cortical regions, respectively), measured via Fluoro-Jade B (upper image set and bar graph), and apoptosis-positive cells (30% and 90% for CA1 and C, respectively), evaluated using the TUNEL assay (lower image set and bar graph) at 5 days of recovery (R5d) in animals treated with QN23 at the onset of reperfusion in a transient global ischemia model using four-vessel occlusion (4VO, see Figure 6c caption). An amount of 2.0 mg/kg was the most effective QN23 dose. * *p* < 0.05 and ** *p* < 0.01, compared to the R5d + vehicle (Vh) by Dunnett’s post-test after ANOVA. (**b**) Neuroprotective and functional outcome effects of QN23 in rats subjected to transient focal ischemic stroke (tMCAO). QN23 administered at 1.5 or 2.5 mg/kg at the onset of the recovery significantly decreased the total infarct volume (54% and 36%, respectively; upper image set and bar graph). * *p* < 0.05 and ** *p* < 0.01, compared to vehicle (Vh) by Dunnett’s post-test after ANOVA. QN23 decreased the neurofunctional score evaluated at 24 or 48 h (lower bar graph). * *p* < 0.05 and ** *p* < 0.01 compared to vehicle (Vh) by Dunn’s post-test after the non-parametric Kruskal–Wallis test. (**c**) Therapeutic window of QN23 in transient global ischemia. QN23 (2.0 mg/kg) significantly decreased cell death (upper image set and bar graphs) and apoptosis (lower image set and bar graphs) in the CA1 and C regions when administered intravenously for up to 3 h of reperfusion after the ischemic episode. * *p* < 0.05 and ** *p* < 0.01, compared to vehicle (Vh) by Dunnett’s post-test, and # *p* < 0.05, by Student’s t test, after ANOVA. (**d**) Study of the long-term effect of QN23 after cerebral ischemia–reperfusion using the neuronal viability assessment. QN23 at 2.0 mg/kg administered at the onset of reperfusion preserved cell integrity in the hippocampal CA1 region, which was similar to the control group when analyzed after 11 weeks of post-ischemia–reperfusion, as quantified in the dot graph. ** *p* < 0.01, compared to the control group by Dunnett’s post-test after ANOVA. (**e**) Study of the long-term effect of QN23 after cerebral ischemia–reperfusion using behavioral tests. QN23 at 2.0 mg/kg administered at the onset of reperfusion preserved animal exploratory activity and spatial memory performances when evaluated after 11 weeks of post-ischemia–reperfusion, which was similar to control animals. * *p* < 0.05, compared to the control group by Dunn’s post-test after the non-parametric Kruskal–Wallis test. Figures were reprinted (adapted) with permission from *Antioxidants*. Copyright 2022 by the authors [45].

The results obtained in these studies encourage the suggestion of QN23, a candidate with an improved neuroprotective effect relative to RP19 and improved pharmacological characteristics, as our new quinolylnitrone lead. Subsequent preclinical studies for the in-depth biological characterization of QN23 were reported in 2022 [45] with a study of the dose–response relationship after intravenous administration (note that previous in vivo experiments (Figure 6 [44]) were carried out with intraperitoneal administration) in both transient global and focal (tMCAO) cerebral ischemia experimental models. The results shown in Figure 7a show that, within the tested range (1.0–2.5 mg/kg), a dose of 2.0 mg/kg was the one that produced the most significant decrease in neuronal death (as observed via Fluoro-Jade B; Figure 7a) and apoptosis-positive cells (as observed via the TUNEL assay; Figure 7a), both in the cortex and CA1 hippocampal regions. A power analysis with the significance level set at 0.05 and statistical power set at 0.8 (80%) determined a sample size of 6 subjects per group, and these studies included 6–7 animals for the QN23-treated groups and 9 animals for the vehicle group. Accordingly, a dose–response study in the tMCAO model, also utilizing intravenous injection, reported 1.5 and 2.5 mg/kg doses as the ones that preserved the neurological performance of the animals subjected to the transient ischemic insult, at both 24 h and 48 h (as observed via the neurodeficit score; Figure 7b), as well as the ones that allowed a significant decrease in infarct size, at both the cortical and subcortical levels (Figure 7b). A power analysis (see above) provided a sample size of six subjects per group, and seven animals were used for each experimental group, except for QN23 4.0 mg/kg, which included five animals because of death within the first 24 h after the procedure. Thus, and in accordance with the results obtained from two independent laboratories, a dose of 2.0 mg/kg was selected for further QN23 characterization [45].

Next, studies addressed the therapeutic window as a relevant aspect of clinical practice. In particular, for acute ischemic stroke, the timing of treatment administration after ischemic insult onset is crucial, especially with regard to the rapid degeneration of the cerebral tissue under blood deprivation. The preclinical assessment of the therapeutic window of new candidates must elucidate the time points at which the treatment exerts its highest effect with the lowest toxicity concerns early in the drug development process. Consequently, treatment administration times ranging from 0 (the onset of reperfusion) to 6 h after the ischemic insult were studied in this report. Neuroprotection was assessed using the previously mentioned Fluoro-Jade B and TUNEL assays, as well as using a neurodeficit score. In this study, the power analysis (see above) rendered 6 subjects per group, with 6 animals used for the QN23- and NXY-treated groups and 10 animals used for the vehicle group. The obtained results (Figure 7c) showed a significant decrease in neuronal degeneration (Fluoro-Jade B) and apoptosis-positive cells when 2.0 mg/kg of QN23 was administered intravenously up to 3 h post-ischemia–reperfusion onset. The functional evaluation results are in accordance with the findings obtained using the staining techniques, achieving a significant reduction in neurodeficit values in the animals treated up to 3 h after the ischemic insult [45].

In addition to the therapeutic window, the increased incidence in younger individuals and extended life expectancy among the eldest necessitate assessing potential sequelae and their modulation using candidate treatments long after the stroke insult. This analysis requires alternative methodological approaches because endogenous mechanisms prevent the observation of cell death and apoptosis produced immediately after the ischemic insult at longer times. Hence, in this report, hippocampal CA1 structural integrity was assessed via the determination of the constitutive cytoplasmic protein S6 in animals recovered 11 weeks after the ischemic insult and treated with 2.0 mg/kg of QN23 at the onset of reperfusion. The power analysis (see above) determined six subjects per group, and six animals were used for each experimental group. The obtained results allow the observation of a significant preservation of hippocampal CA1 structural integrity, with no statistically significant differences from the control experimental group (Figure 7d). Structural analysis was complemented with assessments of exploratory activity and spatial recognition performance, as well as spatial memory tests, 11 weeks after the ischemic insult. Again, in these tests, animals treated with QN23 showed similar performance to the control experimental group (Figure 7e) [45].

Finally, preliminary pharmacological results were also reported by a study of plasma level concentrations and toxicity in control animals. Remarkably, the observed half-lives were very short, ranging from 0.16 h (2.0 mg/kg) to 1.13 h (18 mg/kg). Regarding the toxicity assessment, no systemic signs were detected after a repeated dose administration study for any of the doses, and macroscopic findings could not be observed in macroscopic observations of the organs [45].

**Figure 8:**
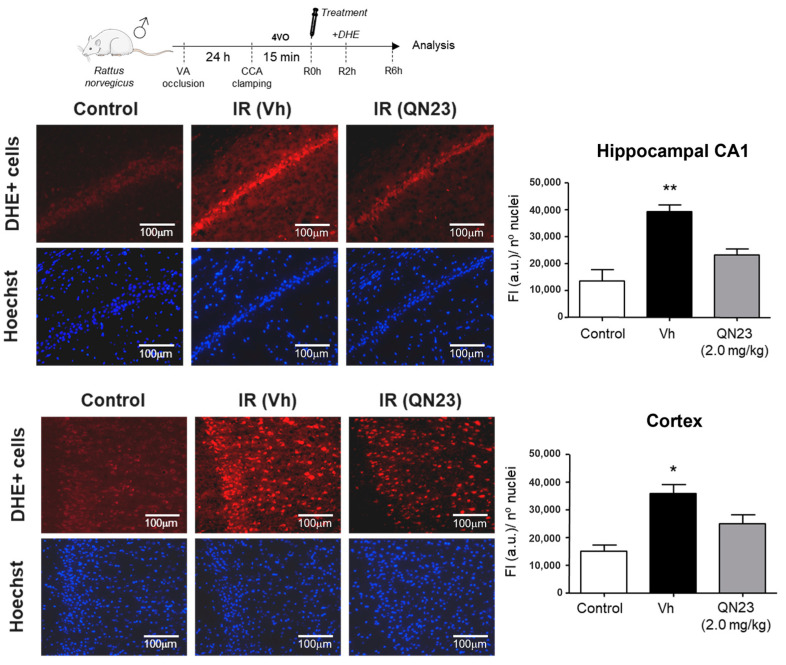
In vivo antioxidant evaluation of QN23. Ischemic animals treated intravenously with QN23 (2.0 mg/kg) at the onset of the reperfusion period after transient global ischemia via four-vessel occlusion (4VO, see Figure 6c caption) presented decreased levels of radical species, as evaluated using a dihydroethidium (DHE) fluorescent probe (3.0 mg/kg) in both hippocampal CA1 and cortical regions. ROS levels evaluated after 6 h of reperfusion (after 4 h of DHE injection) in these regions in animals treated with QN23 (2.0 mg/kg) showed fluorescence intensity values (FI, arbitrary units) similar to those of control (non-ischemic) animals. * *p* < 0.05 and ** *p* < 0.01, compared to the control or QN23-treated group by Newman–Keuls’ post-test after ANOVA. Figure reprinted (adapted) with permission from *Antioxidants*. Copyright 2022 by the authors [45].

Regarding the causes of the observed neuroprotective effect, QN23, as a nitrone-derived drug candidate, is primarily thought to act as a radical scavenger, mainly of hydroxyl radicals (as was determined in an in vitro antioxidant profile panel; Figure 6). Nevertheless, one could refute that these in vitro tests might not properly reflect in vivo conditions and that other mechanisms of action, alternative to radical trapping, could be involved in the neuroprotective effect found for these compounds, as was mentioned in the Introduction Section. In reference [45], the first results of the in vivo antioxidant effect of QN23 were reported, as evaluated using the dihydroethidium (DHE) probe, a fluorescent molecular probe known to react with radical species. These studies included three animals for the control group and five animals for the QN23- and vehicle-treated groups. As shown in Figure 8, animals treated with QN23 showed a decreased DHE fluorescent signal, in both hippocampal CA1 and cortical regions, when compared to vehicle-treated animals, which experienced a dramatic increase in the fluorescent signal due to the ischemic episode and the production of radical species [45]. These results indicate decreased levels of radical species in those ischemic animals treated with QN23, which may indicate an in vivo antioxidant effect.

#### 2.1.3. Future Perspectives: New Quinolyl Derivatives on the Way

**Figure 9:**
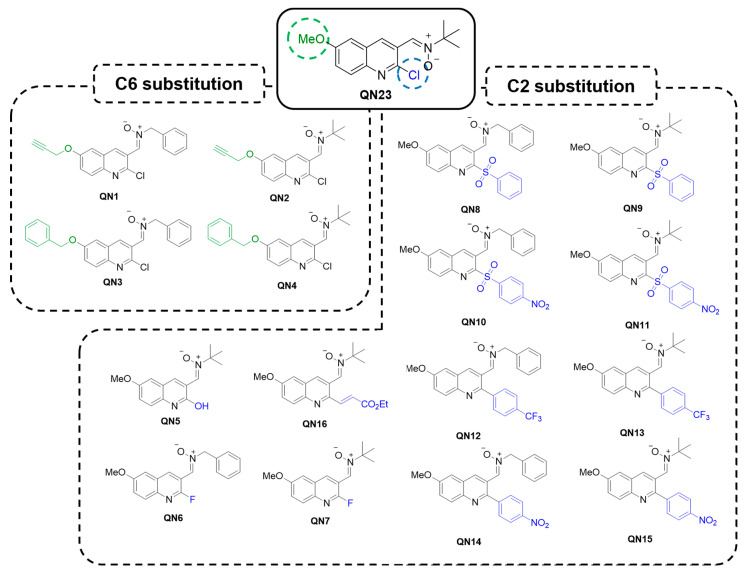
New quinolylnitrones derived from QN23 have been obtained and evaluated in primary neuronal cultures subjected to OGD as potential neuroprotective compounds. The new compounds were obtained based on the modification of the substituents at the C2 and C6 positions in the quinoline core. Compound names are the same as in the original reference. Figure reprinted (adapted) with permission from *Pharmaceuticals*. Copyright 2022 by the authors [46].

Despite reaching the point of identifying QN23 as our most advanced quinolylnitrone, with promising results in the recently reported preliminary preclinical study [45], we continue the research on the quinoline core. New compounds are being developed in collaboration with Dr. Marco-Contelles’ Laboratory in order to find other potentially suitable candidates for the treatment of ischemic stroke, exploring the QN23 core with modifications to the substituents at the C2 and C6 positions, in combination with the *N*-benzyl or *N*-*tert*-butyl, previously proven to be the most effective nitrone groups [46]. The obtained structures are depicted in Figure 9.

Neuroprotection evaluation was reported in our usual screening method based on primary neuronal cultures subjected to OGD. Although no clear correlation between the electronic characteristics and neuroprotective effects could be inferred from this SAR study, some of the new quinolylnitrones were found to exert a protective effect after the OGD insult, as illustrated by the cell viability values, which were superior to the untreated experimental groups. In particular, QN4 and QN15 showed the highest neuroprotection values of the set, along with reasonably extended neuroprotection ranges and no neurotoxicity issues. These compounds had lower EC50 values than QN23 (i.e., a lower concentration is needed to achieve the highest effect), and an improved tolerability range in terms of toxicity and solubility relative to the parental compound [46]. Finally, computational calculations reported a thermodynamically favored reaction in these compounds with the hydroxyl radical [46], as previously reported for QN23 [44].

### 2.2. Nitrone Attachment to Steroids: Cholesterol-Derived Nitrone ISQ201

In parallel to the previous synthetic-oriented screening performed for quinolylnitrones, we started implementing a different approach in which we would attach the nitrone group to steroid-derived cores. In particular, we were aware of the potential neuroprotective effect of progesterone and other steroids on traumatic brain injury [47] and ischemic stroke [48,49]. Steroidal nitrones had been previously documented in the literature [50,51], even though their biological activities were scarcely reported. In our first approach to the steroid–nitrone derivatives, we explored one of their more accessible compounds: cholesterol. Previous examples of cholesterol-derived nitrones, such as 5-ChEPMPO, a cholesteryl ester analog of the spin trap DEPMPO, revealed that the conjugation to cholesterol did not affect the spin trapping properties of the parent nitrone, while it favored membrane-directed action due to the cholesterol fragment [52].

**Figure 10:**
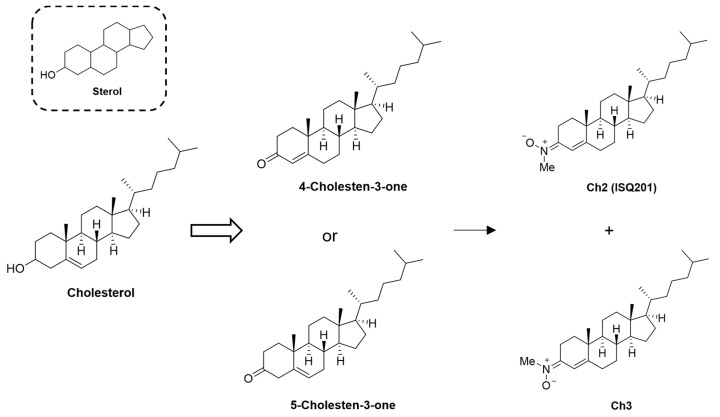
Acquired sterane-derived nitrones from 4-cholesten-3-one or 5-cholesten-3-one, cholesteronitrones 2 (Ch2 or ISQ201) and 3 (Ch3), *E*- and *Z*-isomers, respectively. Reprinted with permission from *J. Med. Chem.* Copyright 2015, the American Chemical Society [53].

Our first biological activity study of cholesterol-derived nitrones, obtained via the attachment of the nitrone moiety to the cholesterol scaffold, was reported in [53]. The synthesis was carried out via *N*-methylhydroxylamine condensation over the commercially available 4-cholesten-3-one or 5-cholesten-3-one starting materials [51,53], which afforded *E*- and *Z*-isomers. In contrast to the rest of the nitrones reported in this review—obtained from the parental aldehyde—these are the only examples of nitrones obtained from the keto-starting material; thus, no isomer was obtained quantitatively over another. In fact, isomers were obtained in relative abundances of 3/1 (*Z/E*) for 4-cholesten-3-one and 1/3 (*Z/E*) for 5-cholesten-3-one, requiring, in both cases, adequate isomer resolution and purification [53]. The two isomeric *N*-methyl cholesteronitrones obtained were named Ch2 (ISQ201, isomer *E*) and Ch3 (isomer *Z*), as depicted in Figure 10.

**Figure 11:**
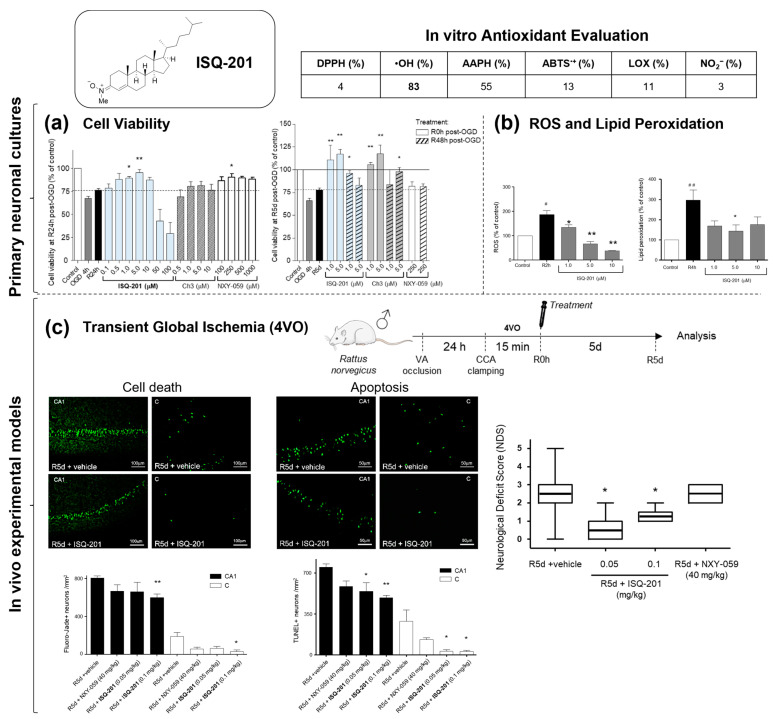
Biological characterization of compound ISQ201 as a candidate for the treatment of ischemic stroke. Table: in vitro antioxidant profile of ISQ201. (**a**) ISQ201 significantly preserved cell viability in primary neuronal cultures exposed to oxygen–glucose deprivation (OGD) when administered in the range of 1.0–5.0 µM at the onset of the recovery period (left bar graph), or when administered at 1.0 µM 48 h after OGD (right bar graph), when compared to the untreated group of cells. * *p* < 0.05 and ** *p* < 0.01, compared to R24h (left graph) or R5d (right graph) by Dunnett’s post-test after ANOVA. (**b**) Effect of ISQ201 on ROS and lipid peroxidation levels in primary neuronal cultures subjected to OGD when administered at the onset of the recovery period. Left bar graph: ISQ201 at 1.0, 5.0 or 10.0 µM significantly decreased ROS levels evaluated at R2h post-OGD recovery. Right bar graph: ISQ201 significantly decreased lipid peroxidation levels evaluated at R4h post-OGD. # *p* < 0.05 and ## *p* < 0.01, R2h or R4h compared to their control by the one-sample *t* test; * *p* < 0.05 and ** *p* < 0.01, compared to R2h or R4h by Dunnett’s post-test after ANOVA. (**c**) Effect of ISQ201 on cell death, apoptosis and the neurological deficit score (NDS) in an experimental model of transient global ischemia in rats via four-vessel occlusion (4VO, see Figure 6c caption). ISQ201 significantly decreased cell death (25% and 90% for hippocampal CA1 and cortical regions, respectively), evaluated via Fluoro-Jade B (left image set and bar graph), and apoptosis-positive cells (33% and 90% for CA1 and C, respectively) using the TUNEL assay (right image set and bar graph) at 5 days of recovery (R5d) in animals treated intraperitoneally with 0.1 mg/kg of ISQ201 at the onset of reperfusion. * *p* <0.05 and ** *p* < 0.01, compared to their respective R5d + vehicle by Dunnett’s post-test after ANOVA. Boxplot: ISQ201 at 0.05 or 0.1 mg/kg also decreased NDS at R5d. * *p* < 0.05, compared to R5d + vehicle by Dunn’s post-test after Kruskal−Wallis’ ANOVA test. Figure reprinted (adapted) with permission from *J. Med. Chem.* Copyright 2015, American Chemical Society [53].

Reactivity and biological characterization in terms of ischemic-stroke-related contributions were documented. Regarding their radical scavenging properties (Figure 11, table), the orbital energy levels, chemical resistance, electronic chemical potential and electrophilicity index were calculated, and all are in accordance with a higher reactivity of ISQ201 (Ch2) against radicals than the corresponding *Z*-isomer (Ch3) [53].

Neuroprotection evaluation in primary neuronal cultures treated at the onset of the recovery period after OGD revealed a neuroprotective range of 0.1–10 µM, with the most potent activity found for 5 µM of ISQ201 (Figure 11a). In contrast, compound Ch3 did not show any significant improvement in cell viability when compared to the untreated experimental group. When the compounds were added 48 h post-OGD, cell viability preservation, measured at 5 days, decreased sharply compared to the experimental groups treated at the onset of the recovery period, even though both compounds, ISQ201 and Ch3, still exerted neuroprotective activity (i.e., higher cell viability values than the vehicle-treated group). This constituted a preliminary approach to the therapeutic window, i.e., the time window in which the addition of a compound still exerts a neuroprotective effect following the ischemic insult, which was later assessed in animal models, as will be discussed in the following paragraphs.

The antioxidant effect of ISQ201 was also determined in primary neuronal cultures subjected to OGD. ROS were measured at 2 h post-OGD recovery, and a significant decrease was observed in the experimental group treated with 5 µM or 10 µM of ISQ201. An assessment of lipid peroxidation was performed 4 h after OGD; similarly, a significant decrease, with similar values to the control group, was observed after the addition of 5 µM of ISQ201 (Figure 11b).

An evaluation of neuroprotection in an animal experimental model of transient global ischemia was determined using the intraperitoneal treatment of ISQ201 at the onset of the reperfusion period. The power analysis (see Section 2.1.2) performed for this study determined a sample size of 6 and 10 subjects in the treated and control groups, respectively. The studies included 6 animals each for the ISQ201- and NXY059-treated groups, and 12 animals for the vehicle group. Analysis of ischemic animals after five days of reperfusion revealed a significant decrease in neuronal death and apoptosis in both hippocampal CA1 and cortical regions, along with an improvement in the neurodeficit score, in the experimental group treated with ISQ201 at 0.05 and 0.1 mg/kg (Figure 11c). Following these results, the preclinical characterization of ISQ201 was further assessed after its intravenous administration, in terms of a dose–response analysis, therapeutic window, long-term effect, infarct size and motor function assays performed, in in vivo models of cerebral ischemia [54].

**Figure 12:**
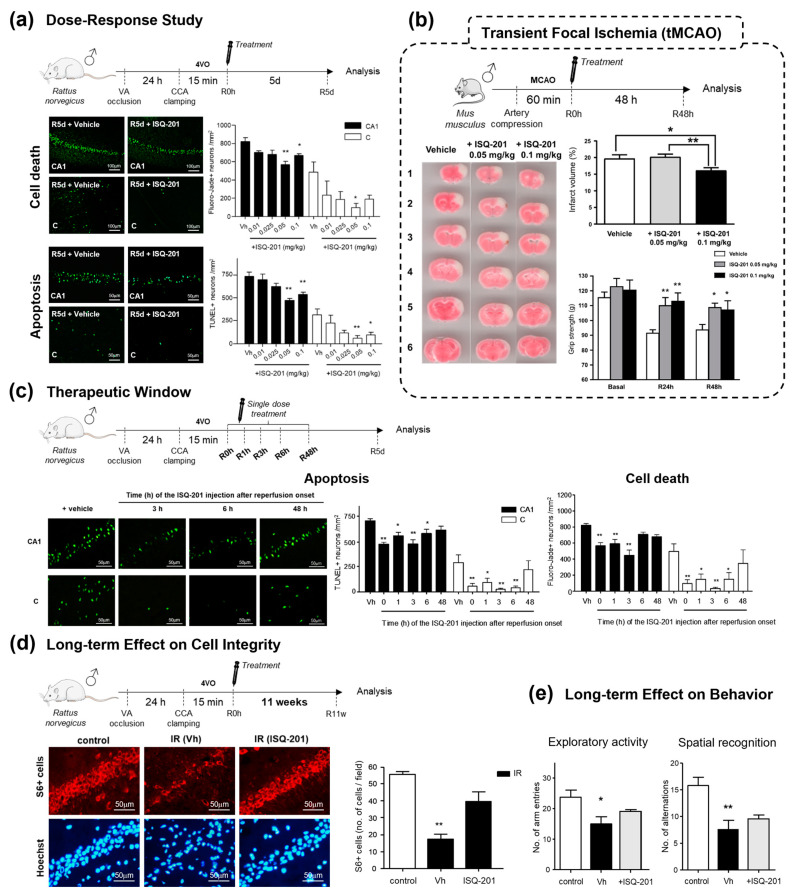
Preclinical characterization of ISQ201. (**a**) Dose–response curve of ISQ201 administered intravenously at the onset of reperfusion in a transient global cerebral ischemia model via four-vessel occlusion (4VO, see Figure 6c caption). ISQ201 significantly decreased cell death (30% and 85% for the hippocampal CA1 and cortical regions, respectively), evaluated via Fluoro-Jade B (upper image set and bar graph), and apoptosis-positive cells (36% and 80% for CA1 and C, respectively) using the TUNEL assay (lower image set and bar graph) at 5 days of recovery (R5d) in animals treated with 0.05 mg/kg of ISQ201, the most effective concentration. * *p* < 0.05 and ** *p* < 0.01, when compared to their respective R5d + vehicle (Vh), by Dunnett’s post-test after ANOVA. (**b**) Neuroprotective and functional outcome effects of ISQ201 in mice subjected to transient focal ischemia (tMCAO). Image set and upper bar graph: ISQ201 significantly decreased the infarct volume (17%) when administered at 0.1 mg/kg at the onset of the recovery period after tMCAO. * *p* < 0.05, compared to vehicle, and ** *p* < 0.01 by Bonferroni’s post-test after ANOVA. Lower bar graph: ISQ201 preserved functional performance, as evaluated using a grip strength test at 24 or 48 h post-tMCAO at 0.05 or 0.1 mg/kg. * *p* < 0.05 and ** *p* < 0.01, compared to vehicle by Bonferroni’s post-test after two-way ANOVA. (**c**) Therapeutic window of ISQ201 in global ischemia. ISQ201 (0.05 mg/kg) significantly decreased apoptosis (image set and left bar graph) and cell death (right bar graph) in CA1 and C regions when administered intravenously up to 6 h of reperfusion after the ischemic episode. * *p* < 0.05 and ** *p* < 0.01, compared to their respective vehicle (Vh), by Dunnett’s post-test after ANOVA. (**d**) Study of the long-term effect of ISQ201 after cerebral ischemia–reperfusion via neuronal viability assessment. ISQ201 at 0.05 mg/kg administered at the onset of reperfusion preserved cell integrity in the hippocampal CA1 region, which was similar to the control group when analyzed after 11 weeks of post-ischemia–reperfusion, as quantified in the bar graph. ** *p* < 0.01, compared to the control group by Dunnett’s post-test after ANOVA. (**e**) Study of the long-term effect of ISQ201 after cerebral ischemia–reperfusion via behavioral tests. ISQ201 at 0.05 mg/kg administered at the onset of reperfusion preserved animal exploratory activity and spatial memory performances when evaluated by performance after 11 weeks of post-ischemia–reperfusion, which was similar to control animals. * *p* < 0.05 and ** *p* < 0.01, compared to the control group using Dunn’s post-test after Kruskal–Wallis’ test. Figures were reprinted (adapted) with permission from *Antioxidants*. Copyright 2020 by the authors [54].

First, the dose–response study included assessing the correct dose among the intravenous administration amounts of 0.01, 0.025, 0.05 and 0.1 mg/kg in a transient global cerebral ischemia model in male rats (Figure 12a). The power analysis (see above) rendered a sample size of six subjects per group. These studies included 6 animals for each ISQ201-treated group and 11 animals for the vehicle group. An assessment of neuroprotection in the cortical and hippocampal CA1 regions via Fluoro-Jade B and the TUNEL assay revealed that doses of 0.05 and 0.1 mg/kg were the most effective in reducing cell degeneration and apoptosis, as well as in preserving functional performance measured via the neurodeficit score after five days of reperfusion [54]. These results are in agreement with those found previously for intraperitoneal administration in the same experimental model [53] and prompted the selection of a dose of 0.05 mg/kg for further characterization.

Therapeutic window analysis, also in the transient cerebral ischemia model, included the evaluation of neuronal death, apoptosis and the neurodeficit score in ischemic animals treated at various time periods after reperfusion onset (0, 1, 3, 6 and 48 h) and assessed after 5 days of reperfusion. The power analysis (see above) determined a sample size of six subjects per group. These studies included 6–7 animals for each experimental group. The results analysis confirmed a significant reduction in neurodegeneration in both cortical and hippocampal CA1 regions when ISQ201 was applied up to 6 h after reperfusion onset (Figure 12c) [54].

Next, a long-term efficacy assessment included the analysis of the structural integrity of the hippocampal CA1 regions after 11 weeks of recovery in animals treated with ISQ201 at the onset of reperfusion. According to the power analysis performed (see above), six animals for each experimental group were included in these studies. The structural integrity was determined using immunohistochemical staining of the constitutive cytoplasmic protein S6. Hippocampal CA1 analysis and cell quantification confirmed a severe loss of cell integrity in the vehicle-treated group and a cell structure preservation in those animals treated with ISQ201 (Figure 12d), which was similar to the control (non-ischemic) animals. Long-term cognitive impairment was studied using functional tests assessing spatial recognition and spatial memory as enduring sequelae. The functional test results confirmed the preservation of a control-like performance in those animals treated with ISQ201 (Figure 12e).

In parallel to these studies of the transient cerebral ischemic stroke model, an independent laboratory carried out a study of a transient focal ischemic stroke model in mice. This was performed via the temporal occlusion of the middle cerebral artery (tMCAO). Animals were intraperitoneally treated with 0.05 or 0.1 mg/kg of ISQ201 after recanalization, and the outcomes—infarct size and grip strength—were measured after 48 h of recovery. For this study, the power analysis (see above) determined eight subjects per group, and nine animals were included for each experimental group. In accordance with the previous results in the transient global ischemia model, either 0.05 mg/kg or 0.1 mg/kg significantly decreased the infarct volume (Figure 12b). Regarding functional status assessed via the grip strength test, and in accordance with infarct volume results, both doses significantly improved the performances of animals in this test, reaching values similar to basal values either 24 h or 48 h after the ischemic insult (Figure 12b).

Finally, preliminary insight into the pharmacology of ISQ201 was explored in healthy animals by analyzing the plasma concentration–time profile after the single bolus intravenous administration of increasing doses of ISQ201 (0.07, 0.25 and 1.00 mg/kg). Linear concentrations vs. time and vs. dose were obtained, and half-lives ranging between 1.5 and 2 h were observed, indicative of a quick and unaltered elimination of the compound [54].

## 3. Discussion

In current clinical practice, only treatments aimed at allowing reperfusion (thrombolysis and thrombectomy) have been granted approval by the FDA or EMA in the treatment of acute ischemic stroke (AIS). However, the relevance of ischemia–reperfusion damage, which further exacerbates ischemic damage and contributes to delayed neuronal death, necessitates the search for new neuroprotective therapies. Numerous approaches have been investigated as potential therapies that are complementary to reperfusion, with the reduction in oxidative stress being one of them.

Nitrone compounds, first developed as radical traps, have gained major relevance in the treatment of oxidative stress-driven diseases due to the ease of obtaining them, their easily modifiable biological activity and their promising results in biological and pharmacological evaluations in experimental models of various diseases.

In this review, we have summarized the development of two families of nitrone-derived compounds in the search for new drug candidates for the treatment of ischemic stroke. These two families were developed according to two different drug development programs. The development of quinolylnitrones is based on a classical medicinal chemistry approach, whereas the development of cholesterol-derived nitrone candidates is based on the exploration of the pharmacological and biological properties of steroid-derived compounds. In either case, we have reached a point at which two lead structures, quinolylnitrone QN23 and cholesteronitrone ISQ201, have shown interesting properties in preliminary preclinical evaluation for the treatment of ischemic stroke.

The driving force of our development was the biological evaluation. Even though radical trapping activity was the original asset of nitrone compounds that prompted their therapeutic suggestion, experimental evidence suggesting complementary modes of action and the multifactorial characteristics of ischemic stroke led us to prioritize evaluation in complex biological settings, such as primary neuronal cultures and animal models of transient cerebral ischemia. Despite this, an analysis of detailed contributions (in vitro antioxidant profile and computational calculations) was also performed and reported as crucial for increasing the knowledge about these compounds.

Regarding the animal experimental models used, they were based on rodent species and focused on transient global and focal cerebral ischemia models. In the transient global ischemia model, there is selective and delayed neuronal death in the so-called “vulnerable areas”, such as the hippocampal CA1 region, usually spanning from 72 h to 7 days [55]. This allows the observation and modulation of cell death mechanisms leading to neuronal apoptosis, which are crucial aspects in the delayed neuronal death observed after the ischemic insult. Additionally, the transient global ischemia model does not present the infiltration of circulating immune cells, which allows researching inflammation-independent contributions to its pathophysiology. All of these experiments were performed according to the ARRIVE guidelines (https://arriveguidelines.org/, accessed on 8 July 2020) regarding comparisons with untreated or control groups, sample size calculations, predefined exclusion criteria, sample randomization, blinded assessments of outcomes and allocation concealment, outcome measures and statistical analysis methods, as well as the appropriate description of the experimental animals and the procedures and results.

We have characterized the effects of QN23 and ISQ201 on the three most clinically relevant aspects in the development of any drug candidate for ischemic stroke according to the STAIR criteria, i.e., (i) the dose–response curve in different experimental models, (ii) the therapeutic window and (iii) the long-term effect. The first studies were carried out with our lead compounds administered at the onset of reperfusion to identify the most effective dose. However, in addition to the dose–response curve necessary to assess the optimal dose to use, delayed treatment application times must be examined for any drug candidate, as often, several hours span between the onset of stroke symptoms and the administration of treatment. The results reported herein confirmed neuroprotective effects for both QN23 and ISQ201 several hours after the reperfusion onset, which contributes to increase the validity of these compounds as drug candidates for ischemic stroke and assist in designing more adequate administration time schedules in future clinical trials. A delayed time of injection ranging from 1 to 6 h post-ischemia was carried out, indicating a significant reduction in damage outcomes with QN23 and ISQ201 injection up to 3 and 6 h, respectively, in our preclinical model, mirroring clinical practice in stroke patients [56]. Furthermore, the administration at the onset of reperfusion is compatible with current recanalization therapies and represents an interesting starting point that may be applicable to humans.

In addition, an assessment of the effect of any drug candidate on long-term-derived sequelae after an ischemic stroke is also crucial due to the increased incidence of ischemic stroke in younger adults with longer life spans. In this regard, we analyzed both the structural integrity and function of the hippocampal CA1 region—previously described as a vulnerable region after a global transient ischemic stroke—in order to assess any potential long-term effects. Remarkably, we observed that both QN23 and ISQ201 preserved the anatomical integrity of this crucial brain region and, thus, allowed the preservation of its proper function, as assessed via spatial memory and exploratory activity performance tests.

Regarding the equivalence of the effective doses found in vitro compared to the administration doses used in rodent-based models, we found a correlation between effective doses in vitro and effective doses in vivo. Thus, ISQ201 showed efficacy at 5 µM in vitro and an effective in vivo dose of 0.05 mg/kg, and QN23 showed efficacy at 100 µM in vitro and its effective in vivo dose was around 2.0 mg/kg. The higher doses of the latter were not a concern for preclinical development, nor the toxicity tests performed. It is noteworthy that doses higher than 100 µM of QN23 were not toxic in in vitro tests either. Nitrones have been described as compounds of low toxicity; in fact, NXY-059, which has been tested in clinical trials, has been assayed in vivo with doses of up to 100 mg/kg [21].

Thus, the results provided by the transient ischemia model have allowed the characterization of QN23 and ISQ201 in the context of delayed neuronal death and neuronal integrity. As a first approach, this allows the description of the molecular pathways implicated in the neuronal component of the neurovascular unit, which agrees with the STAIR criteria recommending the elucidation of the mechanism of action of every cell type affected by the treatment [57]. In addition, the use of transient focal cerebral ischemia models is also covered in the evaluation of QN23 and ISQ201 and the determination of their dose–response curve. Most closely related to the acute ischemic stroke cases registered in the clinic, transient focal ischemia models represent a valuable approach in the protective assessment for stroke-directed compounds in preclinical stages. The transient focal ischemia model was performed via middle cerebral artery occlusion (MCAO) by means of a distal compression model or intraluminal suture. MCA and its ramifications are the most affected in human ischemic stroke; thus, techniques simulating an impairment of MCA-derived circulation are the most adequate [58]. Furthermore, these models reproduce the conditions of the periinfarct area (penumbra) found in human stroke, which is usually the target for neuroprotection therapy and cessation of infarct size extension. In particular, for the filament model used in these studies, it is also valid for the assessment of ischemia–reperfusion damage after induced reperfusion, as it simulates some of the components of the thrombectomy process [59].

In both cases, i.e., the global and focal ischemia experimental models, young adult male rats or mice have been the subject models of this study. As a first approach to the characterization of these compounds, the use of male young adult rats has provided very useful and valid information, in both quantitative and qualitative terms (the results are summarized in Table 1). However, we are aware of the necessity of complementing the characterization of these candidates with comorbidities usually associated with stroke (hypertension and diabetes), as well as with older and female animals. These will be included in our next experimental models, as stated by the STAIR criteria [57], before clinical trials can begin.

The information acquired using the previous experimental models, including the ones performed in primary neuronal cultures, has allowed us to obtain knowledge about the overall effect of the candidates comprising the whole ischemic cascade and its multifactorial components, which was experimentally proven to be beneficial for improved post-stroke recovery. Even though this approach constitutes a main advantage regarding the complexity of stroke and the lack of historical advances in finding a mono-directed therapy [57], it also represents one of the most important limitations in any drug development process, since little information about the mechanism of action is obtained. Precise molecular pathways and the precise cell type affected (neurons, astrocytes, microglia, oligodendrocytes, ependymal cells, white matter, etc.) in the observed protective effect must be considered.

Hence, in parallel to the biological evaluation in experimental models, we carried out some experiments aimed at elucidating the contribution of some main aspects in the nitrone mechanism of action, such as their antioxidant activity. In vivo antioxidant studies with the radical-reactive fluorescent probe DHE have reported a decrease in radical levels with the treatment of QN23 at the onset of the reperfusion period. Despite being unable to assign this effect to any cause at this point, regarding the radical trapping controversy and a possible combined mechanism of action—studies of which are currently conducted—we have confirmed the in vivo antioxidant effect of this molecule in relevant disease experimental models. As a first approach to the elucidation of this candidate’s mechanism of action, this constitutes a promising relevant feature in the fight against ischemic stroke.

Regarding alternative mechanisms of action for ISQ201, special attention can be paid to the cholesterol-derived core. Apart from the probability of driving a particular intracellular localization for this molecule, as reported for similar compounds, such as 5-ChEMPO [60], the cholesterol core probably plays an important role in alternative biological target modulation. The experimental evidence of biological target modulation via neurosteroids suggests a protective effect of these structures [49], an effect that could be shared by the structurally derived ISQ201. Studies aiming to elucidate the precise mechanisms of action of ISQ201 and QN23 are currently being conducted in our lab.

In summary, herein, we have presented an example of a nitrone-derived drug development process for ischemic stroke treatment that has not only led to the identification of two promising candidates but has also allowed, in our opinion, resituating nitrone-derived pharmacology as an interesting field of research to be explored. The versatility and therapeutic suitability of the nitrone group, combined with the almost infinite chemical space, make further research a challenging, but also promising, line of research.

## 4. Summary and Outlook

In this review, we have described the conceptualization, design and evaluation of two promising drug candidates for the treatment of ischemic stroke. Using two different drug development processes, both candidates have been evaluated in clinically relevant experimental models of the disease and showed a safe profile with no side effects or adverse reactions in corresponding toxicity studies. These constitute the first promising approach to a more advanced preclinical evaluation that is currently being performed. As mentioned in the Discussion Section, this will include pharmacological characterization in older and female rodents, as well as an evaluation of stroke-associated comorbidities, such as diabetes or hypertension. Additionally, we are pursuing the elucidation of additional mechanisms of action for QN23 and ISQ201. Herein, the first experimental evidence has reported an in vivo antioxidant profile, even though as the evaluation processes comprised the resulting effect of the ischemic cascade, complementary modes of action in a pleiotropic fashion cannot yet be discarded for QN23 or ISQ201. Altogether, these studies will complement the preclinical characterization reported in this review, covering the required aspects for the eventual launch of these patented drug candidates into clinical trials.

If successful, these compounds would constitute examples of promising contributions to the challenging field of drug development for ischemic stroke. Numerous published studies have addressed the causes of the lack of effective cytoprotective therapies, not only for ischemic stroke but also for other complex diseases [61,62]. In recent decades, the development of highly efficient screening methods based on isolated biological targets, such as high-throughput screening, has shifted back toward common phenotypical screening methods, which were proven to be more adequate in the development of candidates for complex diseases [62,63]. As an example, in our lab, we have devoted great efforts to the optimization of neuroprotection evaluation methods based on primary neuronal cultures subjected to oxygen and glucose deprivation as a cellular model of ischemic insult. Years of development, along with the proper use of experimental controls, have allowed us to develop this phenotypic methodology as a very reliable screening method for every drug candidate for ischemic stroke treatment and to efficiently translate the results into an in vivo experimental model of transient ischemia. Assays carried out by independent laboratories on the effect of the drug candidates identified by our group [42,44,45], using different experimental models, have externally validated our approach. It is noteworthy that, in this evaluation setting, we have not found any ischemic stroke neuroprotective effect for the former candidate NXY-059, which is in agreement with the candidate’s overestimation of preclinical efficacy suggested by several authors after its withdrawal [20].

Therefore, and according to the STAIR criteria [57], we suggest that special attention should be paid to the validation of preliminary evaluation methods, even those at the very first stages of the drug development process, which are crucial for complex diseases, such as ischemic stroke. Although this approach is associated with greater time consumption, resource requirements and the knowledge of the pathophysiology, they are counterbalanced by the higher quality of the entire process and the lower risk of drug withdrawal in advanced steps of drug development, with financial and personal cost savings. Overall, the evident lack of useful therapies, despite their urgent need, necessitates a new consideration of the drug development process in terms of the quality of efficacy and the translation of results.

## 5. Patents

Alberto Alcázar, Emma Martínez, José L. Marco, Mourad Chioua and Juan J. Montoya. Quinolylnitrones for the treatment and prevention of a cerebral stroke or ischemia. U.S. patent US20210330662. E.U. patent EP3863632.

José L. Marco and Alberto Alcázar. Steroidal nitrones for the treatment and prevention of cerebral stroke or ischemia, Alzheimer’s and Parkinson’s diseases, and amyotrophic lateral sclerosis. U.S. patent US10071106. E.U. patent EP3000469.

## Figures and Tables

**Table 1:** Summary of preclinical results.

	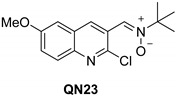	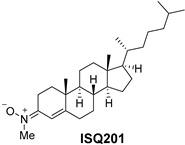
Structural Family	Quinolylnitrones	Cholesteronitrones
Development strategy	SAR-screening-based strategy	Knowledge-based strategy
Screening	Yes	No
SAR	Yes	No
Maximal effective concentration in cellular OGD model	100 µM	5 µM
Effective dose (range) in transient cerebral ischemia	1.5–2.5 mg/kg	0.05–0.1 mg/kg
Therapeutic window	Up to 3 h after reperfusion onset	Up to 6 h after reperfusion onset
Elimination half-life (dose)	0.16 h (2.0 mg/kg)	1.66 h (0.07 mg/kg)

## Data Availability

Data are contained within the article.
